# Association of Bullying Victimization With Suicide Ideation and Attempt Among School-Going Adolescents in Post-conflict Liberia: Findings From the Global School-Based Health Survey

**DOI:** 10.7759/cureus.40077

**Published:** 2023-06-07

**Authors:** Okelue E Okobi, Uzoamaka Egbujo, Jennifer Darke, Amaka S Odega, Obiamaka P Okereke, Olufunmilola T Adisa, Mujeeb A Salawu, Rita Kimble

**Affiliations:** 1 Family Medicine, Medficient Health Systems, Laurel, USA; 2 Family Medicine, Lakeside Medical Center, Belle Glade, USA; 3 Internal Medicine, Lagos University Teaching Hospital, Lagos, NGA; 4 Education, Institute for Teacher Education and Continuing Professional Development (ITECPD), Kumasi, GHA; 5 Education, University of Education, Winneba, Kumasi, GHA; 6 Community and Family Medicine, Windsor University School of Medicine, Basseterre, KNA; 7 Public Health, University of South Wales, Cardiff, GBR; 8 Family Medicine, Olabisi Onabanjo University, Sagamu, NGA; 9 Psychiatry, PsycIME, London, CAN; 10 Family Medicine, Ebonyi State University, Ebonyi, NGA; 11 Internal Medicine and Psychiatry, Lagos State University College of Medicine, Lagos, NGA; 12 Medicine and Surgery, University of Ilorin College of Health Sciences, Ilorin, NGA; 13 Internal Medicine and Psychiatry, Houston Health department, Houston, USA; 14 Research and Development, University of Maryland, Laurel, USA

**Keywords:** liberia, adolescents, suicide attempts, suicide planning, victimization, school bullying

## Abstract

Background

School-based bullying is a global problem that negatively impacts the victims' and perpetrators' health and well-being. There is a paucity of data regarding bullying in schools and its association with suicide behaviors among adolescents in Liberia.

Objective

The study investigated the impact of bullying victimization on suicidal thoughts and suicide attempts among adolescents in Liberia. It aimed to provide insights into the consequences of bullying victimization on adolescents' mental health regarding their thoughts of self-harm and suicide attempts.

Methods

The study utilized data from the 2017 Liberia Global School-based Health Survey (GSHS) to analyze information on 2744 students between the ages of 11 and 18 years, with 52.4% being males. Prevalence rates of bullying victimization and suicide behaviors were calculated using descriptive statistics. Multiple logistic regressions were used to model the relationship between being bullied and experiencing suicidal behaviors (ideation and attempts).

Results

Among the 2744 adolescents examined, 20% experienced suicidal thoughts, with about 30% of the adolescents reporting suicide attempts at some point in the year preceding the survey. Within 30 days prior to the survey, the prevalence of bullying victimization was 50%, with 44.9% experiencing frequent victimization (3 or more days). Bullying victimization was significantly linked to increased odds of suicidal ideation with planning (aOR: 1.86; P < 0.001), at least a suicide attempt (aOR: 2.16; P < 0.001), and multiple attempts at suicide (aOR: 2.67; P < 0.001). We also observed that a greater number of days bullied was dose-dependently associated with higher odds for suicide ideation and attempts.

Conclusion

These findings support and extend those from other developing countries, highlighting the association between school-based bullying and suicidal behaviors. The relatively high prevalence of bullying among adolescents in Liberia underscores the importance of implementing effective anti-bullying policies and suicide prevention strategies in schools.

## Introduction

School-based bullying is a universal issue and has become increasingly recognized as both a public and mental health concern [1]. Among the various forms of school-based violence, bullying is the most prevalent [2]. Bullying is commonly defined as intentionally harmful aggressive behavior that is repetitive and involves an imbalance of power between the perpetrator and the affected person. School bullying may occur in several forms ranging from teasing, name-calling, being rejected, ostracized or excluded from activities, mockery, having belongings taken away, threats, harassment, physical harm, and taunting [3]. 

Previous research have indicated that although bullying victimization is prevalent in adolescents worldwide, there is considerable regional variation in the global prevalence rates of victimization [4,5]. The prevalence of bullying victimization has been reported to be ranging from 30.6% in Southeast Asia [6], 51.3% in Botswana, 18.7% in Australia [7], 10.6% in the United States [8] to 47% in Chile [9]. In a recent study, the pooled prevalence of bullying victimization on one or more days in the past month among 317,869 students who participated in the Global School-based Student Health Surveys (GSHS) in 83 low-income and high-income countries was found to be about 30.5% [10]. The same study also observed that rates of bullying victimization were significantly higher in African countries. Another study involving middle school students in 19 low and middle-income countries also reported high rates of bullying victimization particularly in low-resource settings [11].

Adolescence is a critical period in human social and cognitive development and disruptions during this vital phase of transition have been shown to result in adverse short and long-term psychosocial outcomes [12,13]. Research conducted predominantly in the developed world suggests that there is an association between bullying victimization in adolescence and several adverse health, economic and social outcomes [7,14]. Several studies have reported that bullied students are more likely to skip school, drop out of school, and report poor academic performance [15-17]. Besides these problems, there is robust evidence that links bullying victimization with poor mental health [18]. Bullied adolescents are more likely to exhibit, psychological distress, loneliness, insomnia, anxiety, and depressive symptoms [6,11,19,20]. In addition, negative behaviors such as substance abuse, fighting, and risky sex behaviors have been reported in victimized youths [21]. The effects of bullying extend into adulthood, as there is strong evidence of a significant association between childhood bullying victimization and unemployment, as well as poor physical and mental health outcomes in later life [7,22-25]. To effectively address the negative health and social consequences linked to bullying among adolescents in Sub-Saharan Africa, it is crucial to implement evidence-based intervention and prevention approaches that target distinct risk and protective factors in resource-constrained settings.

One major consequence of bullying victimization that has received considerable attention from researchers recently is suicidal behavior. There is a growing body of literature suggesting that bullying victimization is strongly linked with an elevated risk for suicidal behaviors. Although most of the data on the bully-suicide linkage originates from developed countries [26], there is some consistency from findings in other regions [11,21,27] suggesting that bullying victimization is associated with suicide behaviors (ideation and attempt). Suicide remains one of the leading causes of injury and death among those aged 15-29 years globally [28], and the data shows that about 79% of suicides occur in low-income countries(28). In one study involving 49 low- and middle-income countries (LMICs), 15.3% of adolescents aged 13-15 years had seriously considered suicide in the past year, with those in Africa showing the highest percentage at 19.8% [29].

Despite the disproportionately high prevalence of bullying [10,30] and suicide in low-resource settings like sub-Saharan Africa [31], there is a paucity of research with respect to the association between school-based bullying and suicide among adolescents in Western Africa. So far, studies on adolescent bullying in African countries have mainly focused on the prevalence and correlates of school-based bullying [32-34]. Only a few studies have specifically examined the relationship between bullying victimization and suicide behaviors [35-36]. Given that more than 75% of the world’s adolescent population resides in developing countries, [37], crucial research regarding bullying and its relationship with suicidal behaviors in sub-Saharan adolescents is needed to bridge this gap in the literature.

Liberia, a West African nation that falls under the Anglophone category, is classified as a low-income country with a low human development index (HDI rank of 176). The population of Liberia is approximately 4.8 million, and it has a youthful population structure, with 63% below 25 years old and 32.8% falling within the 10-24 age group. The educational system in Liberia has undergone significant changes and reconstruction following years of civil war and the more recent Ebola outbreak [38-42]. Although there is a potential for these adverse events to influence the well-being, attendance, and academic performance of students [43], no study has explored the prevalence and impact of bullying among school-going adolescents in the country.

Accordingly, we draw on data from the 2017 GSHS survey, a nationally representative sample of Liberian school-going adolescents, to address these gaps by providing a comprehensive overview of the prevalence of bullying victimization in Liberian schools and to examine the association of bullying victimization with suicide behaviors [43-52].

## Materials and methods

Study design and sample

For the present study, a correlational study design was utilized as the most appropriate, as it enabled the researchers to effectively examine the existing relationship between bullying victimization and suicide ideation and attempt among school-going adolescents in post-conflict Liberia. The correlational design is the most appropriate for this study as it not only enables the examination of the existing relationship between two or more different variables but also enables the researchers to observe the natural correlation between the existing variables, in this case, bullying victimization and suicide ideation and attempt. The correlational design also enables the researchers to ask the study participants more with regard to bullying victimization and also understand how it contributes to suicide ideation and attempts among students.

For the present study, data were collected from the GSHS database. The GSHS is a publicly available database, a cross-sectional survey conducted in interested WHO member states to assess the behavioral risk factors and protective factors in multiple areas among youth in school [43-52]. The United States Centers for Disease Control and Prevention (CDC) provides technical assistance. Data were collected by means of a self-administered questionnaire. More information on the methodology and the topics addressed by the survey are available on the WHO website. The GSHS questionnaire in each country consists of validated survey items that assess behavioral risk factors and protective factors across various domains among adolescents attending secondary school. Ethical approval and necessary permissions are obtained from relevant authorities by WHO or its delegated agency before collecting data from a nationally representative sample of students. The students voluntarily participate by recording their responses on a computer-scannable form provided during a standard class period. The survey ensures anonymity as no personally identifiable information is collected. Approximately two years later, clean data files, devoid of individual- or school-level identifiers, are publicly accessible. When analyzing this cross-sectional data, we have adhered to the recommendations outlined in Strengthening the Reporting of Observational Studies in Epidemiology (STROBE). Also while analyzing the GSHS dataset, we did not need an IRB approval number as analyzing publicly available data sets typically does not require IRB approval, following standard ethical research guidelines. The publicly available data used in this research are not individually identifiable and do not involve human subjects.** **

Sampling

A two-stage approach is employed in GSHS-participating countries to obtain a nationally representative sample of school children in the target age ranges. Initially, schools are randomly selected using a probability proportionate to size (PPS) method, ensuring geographic diversity. Subsequently, classrooms with a high proportion of students from the targeted age groups are sampled within each participating school. This sampling process ensures an equal chance of selection for every student. In this study, participants consisted of grades 7-12 students from selected schools in Liberia. Each student record was assigned numerical weights to generalize the findings to the eligible population. The study achieved a high participation rate, with 98% of sampled schools and a student response rate of 73%, resulting in an overall response rate of 71%.

Inclusion and exclusion criteria

The inclusion criteria entailed individuals aged between 10 and 19 years, given that adolescents are considered to be persons aged 10-19 years. The participants had to be enrolled in a Liberian school at the time of the study and had to have experienced bullying in school. Further, the inclusion criteria stipulated that the participants had to be Liberian citizens.

The exclusion criteria entailed students below the age of 10 years and those above 19 years of age. Further, adolescents who had not experienced suicide were excluded from the study, alongside those who were not enrolled in any school at the time of stud. Additionally, adolescent students who were not citizens of Liberia were also excluded from the study.

Measures

Exposure Variable

The exposure variable (see Table 1) of interest was bullying victimization, and this was assessed in three ways i) any bullying victimization, ii) type of bullying victim­ization, and iii) frequency of bullying vic­timization. In the GSHS, each of these variables was a single-item measure. Bullying was defined in the survey as “when a student or group of students say or do bad and unpleasant things to another student” or as “when a student is teased a lot in an un­pleasant way or when a student is left out of things on purpose.” It was explained that bullying was not “when two students of about the same strength or power argue or fight” or “when teasing is done in a friendly and fun way." Specifically, bully victimization and frequency of bullying victimization were assessed by the question: “During the past 30 days, on how many days were you bullied?” Response options included 0 days, 1 or 2 days, 3 to 5 days, 6 to 9 days, 10 to 19 days, 20 to 29 days, and all 30 days. This question was a modified version of the item assessing victimization frequency from the widely used Olweus Bully/Victim Questionnaire. To determine any bullying victimization, students who were not bullied were coded as 0, whereas students who were bullied at least on one occasion were coded as 1. Based on previous research and the skewness of the distribution, the frequency of victimization was coded as: non-victimization (0 days), occasional victimization (1 or 2 days), and frequent victimization (3 or more days). Finally, the item "type of bullying victimization" was assessed by the single item “during the past 30 days, how often were you bullied the most?” Response options were further classified as physical bullying victimization (“I was hit, kicked, pushed, shoved around, or locked indoors”), nonphysical bullying victimization (“I was made fun of because of my race, nationality, or color,” “I was made fun of because of my religion,” “I was made fun of with sexual jokes, comments, or gestures,” “I was left out of activity on purpose or completely ignored,” or “I was made fun of because of how my body or face looks”), and unspecified bullying victimization (“I was bullied in some other way”).

**Table 1:** Summary of the codifying systems for exposure variable of interest.

Measure	Description
Bullying Victimization	The exposure variable of interest, assessed in three ways:
	i) Any bullying victimization
	ii) Type of bullying victimization
	iii) Frequency of bullying victimization
	Each of these variables was a single item measure in the GSHS
Definition of Bullying	Bullying was defined in the survey as "when a student or group of students say or do bad and unpleasant things to another student" or as "when a student is teased a lot in an unpleasant way or when a student is left out of things on purpose." It was explained that bullying was not "when two students of about the same strength or power argue or fight" or "when teasing is done in a friendly and fun way".
Frequency of Bullying Victimization	Assessed by the question: "During the past 30 days, on how many days were you bullied?" Response options included:
	- 0 days
	- 1 or 2 days
	- 3 to 5 days
	- 6 to 9 days
	- 10 to 19 days
	- 20 to 29 days
	- All 30 days
	This question was a modified version of the item assessing victimization frequency from the Olweus Bully/Victim Questionnaire.
Coding of Bullying Victimization	To determine any bullying victimization, students who were not bullied were coded as 0, whereas students who were bullied at least once were coded as 1.
	Frequency of victimization was coded as follows:
	- Non-victimization (0 days)
	- Occasional victimization (1 or 2 days)
	- Frequent victimization (3 or more days)
Type of Bullying Victimization	Assessed by the single item: "During the past 30 days, how often were you bullied the most?" Response options were further classified as:
	- Physical bullying victimization ("I was hit, kicked, pushed, shoved around, or locked indoors")
	- Nonphysical bullying victimization ("I was made fun of because of my race, nationality, or color," "I was made fun of because of my religion," "I was made fun of with sexual jokes, comments, or gestures," "I was left out of activity on purpose or completely ignored," or "I was made fun of because of how my body or face looks")
	- Unspecified bullying victimization ("I was bullied in some other way")

Outcome Variables

For this analysis (see Table 2), three main outcome measures were selected from the data: suicidal ideation, at least one suicide attempt, and multiple suicidal attempts. Each of these three outcome variables was a single-item measure. Specifically, the item “during the past 12 months, did you ever seriously consider attempting suicide?” was used to assess suicidal ideation. The responses were dichotomized as “yes” (1) or “no” (0). A suicidal attempt was measured with the question “during the past 12 months, how many times did you actually attempt suicide?” The responses to this question were “0”, “1”, “2 or 3”, “4 or 5”, and “6 or more times”. We further categorized these responses into two outcomes. In line with previous secondary analyses of the GSHS data in sub-Saharan Africa, we utilized a binary recoding approach for the variable related to suicidal attempts; no attempt (0) and one or more attempts (1) for analysis. Second, to ascertain those with multiple suicide attempts, we also dichotomized the suicide attempt variable into (0) no suicide attempt and (1) having two or more attempts based on previous research. See Tables 1 and Table 2 for summaries of the data coding systems of the exposure and outcome variables, respectively.

**Table 2:** Summary of the codifying systems for outcome variable of interest.

Variable	Description
Suicidal Ideation	Single-item measure assessed using the question: "During the past 12 months, did you ever seriously consider attempting suicide?" Responses were dichotomized as "yes" (1) or "no" (0)
Suicidal Attempt	Single-item measure assessed using the question: "During the past 12 months, how many times did you actually attempt suicide?" Responses:
	- "0"
	- "1"
	- "2 or 3"
	- "4 or 5"
	- "6 or more times"
	Responses were categorized into two outcomes for analysis:
	- "No attempt" (0)
	- "One or more attempts" (1)
Multiple Suicide Attempts	Dichotomized variable based on the suicide attempt variable:
	- "No suicide attempt" (0)
	- "Having two or more attempts" (1)

Control Variables

The selection of exposure variables for the study on suicide behaviors in adolescents within sub-Saharan African countries was based on previous research. Demographic variables considered included gender, age, and school grade. Control factors such as family, school, and personal lifestyle were also included to assess the predictive impact of bullying victimization on suicide outcomes, including suicide ideation, at least one suicide attempt, and multiple suicide attempts. For detailed information on the variables, survey questions, and coding used for statistical analysis, please refer to Table 3.

**Table 3:** 30-day prevalence of bullying victimization (frequency and method of bullying)

Variable	Overall, %, (95% CI)	Male, %, (95% CI)	Female, %, (95% CI)
Bullying victimization			
At least on one day	50.0 (48.0 – 52.0)	45.8 (43.0 – 48.6)	53.4 (50.4 – 56.4)
Bullying frequency			
Occasional victimization (1 or 2 days)	55.1 (52.3 – 58.0)	55.3 (51.1 – 59.4)	57.0 (52.8 – 61.0)
Frequent victimization (3 or more days)	44.9 (42.0 – 47.7)	44.7 (40.6 – 48.9)	43.0 (39.0 – 47.2)
Bullying method			
Physical	21.0 (18.5 – 23.8)	22.1 (18.4 – 26.2)	19.7 (16.3 – 23.7)
Non-physical	49.4 (46.2 – 52.6)	52.3 (47.6 – 56.9)	46.9 (42.3 – 51.5)
Unspecified	29.6 (26.8 – 32.6)	25.7 (21.8 – 30.0)	33.4 (29.2 – 37.9)

Statistical Analyses

Analyses were performed with Stata 14.0 statistical software (StataCorp LP, College Station, Texas, USA). Sample weights were used in all analyses, so the results are generalizable to the population. We utilized descriptive statistical methods to provide prevalence and demographic estimates. Next, Pearson chi-square was used to test the association between bullying victimization variables and demographic characteristics. The multivariate analysis involved the use of binary and hierarchal logistic regressions to examine the association between all the bullying victimization variables and each suicide outcome while simultaneously adjusting for other factors. The results from the regression analyses are presented as odds ratio (OR) and 95% confidence interval (CI). Bonferroni correction was applied to correct for multiple comparisons (0.05/9 = 0.005) and statistical significance was defined as a two-tailed p-value of < 0.005.

## Results

Sample characteristics

A total of 2,744 school-going adolescents in Liberia were included in the study. Of these, 52.4% were males, and 56.3% were grades 7 to 9 students. Approximately 50% of students reported at least one form of bullying in the past 30 days. Of these, 55.1% reported occasional bullying (1 or 2 days), and 44.9% experienced frequent bullying (3 or more days) in the month preceding the survey. Almost half of those who reported being bullied (49.4%) in the past 30 days experienced a non-physical form of bullying. Among adolescents, 20% experienced thoughts of suicide in the previous 12 months, while 30% made suicidal attempts during the same period. Of those who attempted suicide, 16.5% had a single episode, while 17.2% made multiple attempts within the previous 12 months. The general distribution of the variables examined and stratified by gender is presented in Table 4. There were significant gender differences across the following factors: age, alcohol drunkenness, food security, and close friends.

**Table 4:** Sample distribution by gender χ2   = chi-square

Variable	Total n (%)	Males n (%)	Females n (%)	χ^2^	p-value
Demographics					
Age (in years)				4.38	0.036
≤ 17 years	1277 (49.6)	644 (47.7)	633 (51.8)		
≥ 18 years	1296 (50.4)	707 (52.3)	589 (48.2)		
School grade				3.17	0.075
Grade 7 to 9	1467 (56.3)	745 (54.6)	722 (58.0)		
Grade 10 to 12	1142 (43.8)	620 (45.4)	522 (42.0)		
Personal factors					
Alcohol drunkenness				9.81	0.002
Yes	476 (20.0)	282 (22.4)	194 (17.3)		
No	1907 (80.0)	977 (77.6)	930 (82.7)		
Leisure-time sedentary behaviour				3.07	0.080
≥ 3 hours/day	477 (20.1)	231 (18.8)	246 (21.7)		
< 3 hours/day	1890 (79.9)	1000 (81.2)	890 (78.3)		
Cannabis use				1.71	0.191
Yes	178 (7.7)	102 (8.4)	76 (6.9)		
No	2132 (92.3)	1113 (91.6)	1019 (93.1)		
School factors					
Physical attack				0.04	0.849
Yes	1457 (56.9)	773 (57.1)	684 (56.7)		
No	1103 (43.1)	581 (42.9)	522 (43.3)		
Truancy				2.97	0.085
Yes	1087 (45.8)	585 (47.5)	502 (44.0)		
No	1287 (54.2)	647 (52.5)	640 (56.0)		
Peer support				2.17	0.141
Yes	858 (36.9)	464 (38.3)	394 (35.3)		
No	1469 (63.1)	748 (61.7)	721 (64.7)		
Close friends				6.85	0.009
Yes	2217 (87.6)	1181 (89.2)	1036 (85.8)		
No	315 (12.4)	143 (10.8)	172(14.2)		
Family factors					
Parental supervision				0.48	0.487
Yes	1082 (45.7)	570 (46.4)	512 (45.0)		
No	1284 (54.3)	658 (53.6)	626 (55.0)		
Parental understanding				0.09	0.768
Yes	961 (41.3)	503 (41.6)	458 (41.0)		
No	1365 (58.7)	706 (58.4)	659 (59.0)		
Parental bonding				2.51	0.113
Yes	877 (38.4)	437 (36.9)	440 (40.0)		
No	1405 (61.6)	748 (63.1)	657 (60.0)		
Parent intrusion of privacy				1.71	0.191
Yes	465 (19.8)	232 (18.8)	233 (21.0)		
No	1879 (80.2)	1001 (81.2)	878 (79.0)		
Food security				8.42	0.004
Yes	426 (16.7)	251 (18.8)	175 (14.5)		
No	2118 (83.3)	1085 (81.2)	1033 (85.5)		
Suicide behaviours					
Suicide ideation				0.72	0.397
Yes	647 (26.2)	333 (25.5)	314 (27.0)		
No	1822 (73.8)	973 (74.5)	849 (73.0)		
Suicide attempt (at least 1 attempt)				0.04	0.843
Yes	826 (33.6)	439 (33.8)	387 (33.4)		
No	1633 (66.4)	861 (66.2)	772 (66.6)		
Multiple suicide attempts				1.52	0.217
Yes	419 (17.0)	233 (17.9)	186 (16.1)		
No	2040 (83.0)	1067 (82.1)	973 (83.9)		

Bivariate association between bullying victimization, demographics, and suicide behaviors

Significant bivariate associations were observed between bullying victimization, gender, and school grade. Specifically, as shown in Table 5, 45.8% of males compared to 53.4% of female students reported bullying at least once in the past 30 days (χ2 = 13.17; p-value <0.001). Also, about 52.7% of students in grades 7 to 9 reported bullying compared to 46.4% of students in grades 10 to 12 (χ2 = 8.97; p-value = 0.003). No significant association was observed between age and bullying victimization. Further, we found significant associations between bullying victimization and all three domains of suicide behaviors (suicide ideation, at least one suicide attempt, and multiple suicide attempts) in our bivariate analysis. About six in 10 students that were bullied (62.0%) compared to four in 10 students that were not bullied (38.0%) reported suicidal ideation (χ2 = 52.95; p-value <0.001). Similarly, having been bullied was associated with a higher likelihood of reporting at least one suicide attempt (68.0% vs. 39.6%; χ2 = 160.5; p-value <0.001), as well as reporting multiple suicide attempts (75.7% vs. 43.7%; χ2 = 129.86; p-value <0.001) during the previous 12 months than those who were not bullied.

**Table 5:** Bivariate association between bullying victimization (once or more), demographic variables, and suicide behaviors

Variable	Bullying victimization (1 or more days) n = 1177			
	No	Yes	χ^2^	p-value
	n (%)	n (%)		
Demographics				
Gender			13.17	<0.001
Male	651 (54.2)	550 (45.8)		
Female	494 (46.6)	567 (53.4)		
Age (in years)			0.53	0.466
≤ 17 years	573 (50.5)	562 (49.5)		
≥ 18 years	567 (49.0)	591 (51.0)		
School grade			8.97	0.003
Grade 7 to 9	612 (47.3)	682 (52.7)		
Grade 10 to 12	550 (53.6)	477 (46.4)		
Suicide behaviours				
Suicide ideation			52.95	<0.001
Yes	224 (38.0)	365 (62.0)		
No	897 (55.5)	718 (44.5)		
Suicide attempt (at least 1 attempt)			160.5	<0.001
Yes	240 (32.0)	511 (68.0)		
No	888 (60.4)	583 (39.6)		
Multiple suicide attempts			129.86	<0.001
Yes	93 (24.3)	290 (75.7)		
No	1035 (56.3)	804 (43.7)		

Multivariate logistic regression for the association between bullying victimization, suicidal ideation, and suicide attempt

Using logistic regression models adjusted for age, gender, school grade, and other covariates, as depicted in Table 6, bullying victimization in the previous 30 days was significantly associated with suicidal ideation (aOR: 1.86; 95% C.I: 1.41 - 2.46; p-value <0.001), at least one suicide attempt (aOR: 2.16; 95% C.I: 1.66 - 2.80; p-value <0.001), and multiple suicide attempts (aOR: 2.67; 95% C.I: 1.85- 3.84; p-value <0.001). Overall, not all bullying types were associ­ated with similarly increased odds for all measures of suicide ideation and behav­ior. Specifically, compared with no bullying victimiza­tion, reporting nonphysical bullying victimization was associated with significantly greater odds of suicidal ideation (aOR: 1.76; 95% C.I: 1.25- 2.48; p-value <0.001); at least one suicide attempt (aOR: 1.84; 95% C.I: 1.32- 2.55; p-value <0.001); and multiple suicide at­tempts (aOR: 2.10; 95% C.I: 1.40- 3.14; p-value <0.001). Also, when compared to no bullying victimization, physical bullying victimization was associated with increased odds of suicidal ideation (aOR: 1.51), at least one suicide attempt (aOR: 1.79), and multiple suicide attempts (aOR: 1.72); however, these fell slightly short of statistical significance after correcting for multiple comparisons.

**Table 6:** Adjusted Logistic regression models for the association between bullying victimization and suicide ideation and attempts. All models were adjusted for demographic variables (age, gender, and school grade), personal and lifestyle factors (leisure-time sedentary behavior, alcohol drunkenness, and cannabis use), school environment factors (truancy, physical attack, close friends, and truancy), and family-related factors (parent monitoring, parent bonding, parent intrusion of privacy, parent understanding, and food security);  aOR = adjusted Odds ratio; β = regression co-efficient; CI = confidence interval

Variables in models	Suicidal ideation (n= 682)				At least one suicide attempt (n= 861)				Multiple suicide attempts (n= 439)			
	β	aOR	95% CI	p-value	β	aOR	95% CI	p-value	β	aOR	95% CI	p-value
Any bullying victimization in past 30 days												
No (reference)		1.00				1.00				1.00		
Yes	0.620	1.86	1.41, 2.46	<0.001	0.768	2.16	1.66, 2.80	<0.001	0.980	2.67	1.85, 3.84	<0.001
Frequency of bullying victimization in past 30 days												
No victimization (reference)		1.00				1.00				1.00		
Occasional victimization (1 or 2 days)	0.581	1.79	1.31, 2.44	<0.001	0.700	2.01	1.50, 2.70	<0.001	0.670	1.95	1.29, 2.97	0.002
Frequent victimization (3 or more days)	0.689	1.99	1.38, 2.86	<0.001	0.884	2.42	1.72, 3.40	<0.001	1.41	4.08	2.65, 6.26	<0.001
Most common type of bullying victimization in past 30 days												
None		1.00				1.00				1.00		
Physical bullying	0.411	1.51	0.93, 2.44	0.094	0.580	1.79	1.14, 2.80	0.012	0.543	1.72	0.98, 3.01	0.057
Non-physical bullying	0.563	1.76	1.25, 2.48	0.001	0.608	1.84	1.32, 2.55	<0.001	0.740	2.10	1.40, 3.14	0.001
Unspecified	0.287	1.33	0.89, 2.01	0.168	0.404	1.50	1.02, 2.19	0.037	0.095	1.10	0.64, 1.88	0.729

Furthermore, bullying victimization frequency showed an increasing exposure-re­sponse effect, with a higher number of days of victimization associated with greater odds of suicidal ideation and be­havior. Compared with no bullying victim­ization, reporting occasional bullying victimization (of 1 or 2 days ) was associated with signifi­cantly increased odds of suicidal ideation (aOR: 1.79; 95% C.I: 1.31- 2.44; p-value <0.001), at least one suicide attempt (aOR: 2.01; 95% C.I: 1.50- 2.70; p-value <0.001), and multi­ple suicide attempts (aOR: 1.95; 95% C.I: 1.29- 2.97; p-value = 0.002). Frequent bullying victimization (of 3 or more days) was associated with significantly greater odds of sui­cidal ideation (aOR: 1.99; 95% C.I: 1.38- 2.86; p-value <0.001), at least one suicide attempt (aOR: 2.42; 95% C.I: 1.72- 3.40; p-value <0.001), and multiple suicide attempts (aOR: 4.08; 95% C.I: 2.65- 6.26; p-value <0.001).

## Discussion

Drawing on a large nationally representative dataset from Liberia, this is among the first studies to investigate the link between bullying victimization and suicidal ideation and suicide attempt among a non-clinical sample of adolescents. This study identified three main findings: first, about 20% of adolescents reported having had thoughts about suicide, 16.5% made at least one attempted suicide at some point during the past year, and about 17.2% made multiple suicide attempts within the past year; second, approximately half (50%) of the adolescents were bullied during the past 30 days; third, bullying victimization was significantly associated with suicidal ideation with planning and suicide attempt.

Compared to the range of estimates of bullying victimization among adolescents from studies in other sub-Saharan developing countries [35,36,44], the estimates of bullying victimization in our study are substantially higher. While the reasons for the disproportionately elevated rates of bullying victimization in school-going adolescents in Liberia cannot be obtained from our data, one potential explanation could be the country’s history of violence, wars, and conflict [42]. Such a hostile environment may possibly cause downstream and distal effects in adolescents' behaviors who may engage in violence and bullying as a way of coping. Importantly, the evidence of higher estimates of bullying victimization and the pioneering nature of the present study emphasizes and points to the need for further evidence through rigorous qualitative and epidemiological research to clarify the extent of bullying victimization and identify potential anti-bullying strategies that may be implemented in schools in Liberia.

Our findings showed that bullying victimization is associated with suicidal behavior among school-going adolescents. This finding matches the observation from previous studies where bullying was demonstrated to be independently related to suicidal risks and behavior among children [35,45,46]. Additionally, our analysis indicates a potential dose-response relationship between bullying and suicide behaviors as we found that adolescents who were more frequently bullied had greater odds of suicide planning and attempts than those that were bullied less frequently or never at all. Beyond this association, a related and important finding from our study that warrants serious attention is that amongst the forms of bullying victimization, non-physical bullying emerged as the only form of bullying that was significantly linked to higher odds of suicide behaviors [6, 8]. One form of non-physical bullying victimization is cyberbullying. Cyberbullying has been defined as the decisive attempt to cause harm to another individual or being mean to another individual online, on social media, and in group texts or texts. Adolescents and younger persons targeted by cyberbullies face the risk of adverse mental health effects, including depression, anxiety, and suicide ideation and attempts. Recent studies have indicated that individuals who experience cyberbullying are over four times as prone to reporting suicidal thoughts and attempts compared to those who do not [45-53]. Thus, the present study's result implies that interpersonal problems and social difficulties such as non-physical bullying can induce feelings of distress and shame, which can negatively affect adolescents’ mental health and increase their susceptibility to suicide behaviors.

Study implications and recommendations

Consistent with the stress process theory [47], this study found a significant association between bullying and suicide behaviors. The high rates of bullying and its association with suicide risk among school-going adolescents have some implications for mental health intervention and prevention efforts, school health, and policy directions in Liberia. Clinicians should collaborate with school authorities and teachers to offer education on the recognition of bullying and early warning signs of poor mental health among adolescents and provide referrals to specialist mental health services. Targeted suicide and anti-bullying prevention and treatment strategies would rely on a multi-disciplinary team approach, especially given the fragile mental health infrastructure available to youths in Liberia [48]. Relatedly, given the unavailability of robust adolescent mental health systems in Liberia, our results support the need to develop school mental health programs as potentially an effective way to empower adolescents and reduce the burden of adolescent suicide [49-51].

Limitations

The findings of this study should be interpreted considering some methodological and contextual limitations. First, we relied on data from a cross-sectional survey. Thus, our findings are limited to associations, and causal inferences cannot be drawn. Also, due to the cross-sectional nature of the study, we were unable to control for other theoretical factors such as mental health history, prior history of suicide attempts, and family factors which may influence suicidal behaviors among the study population. Future studies should employ longitudinal designs to evaluate the impact of bullying victimization on youth suicide in Liberia. Second, the self-report nature of the study variables has the potential to introduce recall and social desirability bias. Third, our data sample was limited to only school-going adolescents and did not account for out-of-school adolescents or home-schooled adolescents. Therefore, taken together, these factors may have biased our results and should be considered when interpreting the findings.

## Conclusions

School-going adolescents in Liberia are prone to substantially high rates of bullying. The prevalence of suicidal thoughts among the studied adolescents was 20%, and approximately 30% reported suicide attempts in the year prior to the survey. Within the 30 days preceding the survey, bullying victimization affected 50% of the adolescents, with 44.9% experiencing frequent victimization. The study found a significant association between bullying victimization and increased odds of suicidal ideation with planning, at least one suicide attempt, and multiple attempts at suicide. Additionally, a higher frequency of bullying was dose-dependently linked to elevated odds of suicidal ideation and attempts. Based on the study's findings, we recommend the following actions to address the issue of bullying and its association with suicidal ideation/attempts. First, implement comprehensive anti-bullying programs in schools, emphasizing prevention, early intervention, and the creation of a safe and supportive environment for students. Second, enhance mental health support services within educational settings by increasing the availability of counselors, psychologists, and resources for timely intervention and support. Additionally, foster awareness and education through campaigns targeting students, parents, teachers, and the community to increase understanding, empathy, and early identification of bullying situations. Moreover, strengthening collaboration between schools and mental health professionals through training sessions, workshops, and effective communication channels can enhance the identification and support of students facing bullying and suicidal behaviors. Finally, conducting longitudinal studies and further research is recommended to track the long-term effects of bullying on mental health and explore underlying mechanisms and risk factors to inform targeted prevention strategies.
